# The association between academic stress, social support, and self-regulatory fatigue among nursing students: a cross-sectional study based on a structural equation modelling approach

**DOI:** 10.1186/s12909-022-03829-2

**Published:** 2022-11-14

**Authors:** Zhang Yuhuan, Zheng Pengyue, Chen Dong, Niu Qichao, Pang Dong, Song Anqi, Jiang Hongbo, Di Zhixin

**Affiliations:** 1 The Second Affiliated Hospital of Harbin Medical University, Student Department, Internship Researcher, 246 Xuefu Road, Heilongjiang Province Harbin, 150086 China; 2 Heilongjiang University of Chinese Medicine, 24 Heping Road, Harbin, Heilongjiang Province 150040 China; 3 Heilongjiang Nursing College, Advanced Practice Nurse, Comprehensive Department of Nursing Education and Research, 209 Xuefu Road, Harbin, Heilongjiang Province 150086 China; 4 Student Department, the Second Affiliated Hospital of Harbin Medical University, 246 Xuefu Road, Harbin, Heilongjiang Province 150086 China; 5 Department of Neurology, The Second Affiliated Hospital of Harbin Medical University, 246 Xuefu Road, Harbin, Heilongjiang Province 150086 China; 6 Department of Ultrasound Medicine, The Second Affiliated Hospital of Harbin Medical University, 246 Xuefu Road, Harbin, Heilongjiang Province 150086 China

**Keywords:** Social support, Academic pressure, Self-regulatory fatigue, Mediating effect

## Abstract

**Background:**

Emphasizes the state of academic stress, social support, and self-regulatory fatigue on the physical and mental development of Chinese nursing students, the purpose of this study was to investigate the relationship between these variables and the mediating role of social support in academic stress and self-regulatory fatigue among a group of undergraduate nursing students in Heilongjiang Province, China, in order to provide a theoretical basis for working to reduce nursing students’ self-regulatory fatigue.

**Methods:**

In this cross-sectional study, 1703 nursing students from various academic years completed the scales of social support, academic stress, and self-regulatory fatigue. In the end, there were 797 valid questionnaires, for a recovery rate of 46.80%. For statistical analysis, the independent t-test, Kruskal Wallis test, and Pearson correlation coefficient were used. In addition, we undertake analyses using structural equation modeling.

**Results:**

The bulk of nursing students, or 81.4%, are between the ages of 19 and 21. Eighty percent were females. The bulk (93.0%) was comprised of freshmen. Academic stress, social support, and self-regulatory fatigue had total scores of 111.28 ± 29.38, 37.87 ± 6.70, and 45.53 ± 5.55,respectively. Academic stress was correlated with social support and self-regulatory fatigue (all *p* < 0.001). Social support was an intermediate variable (*p* < 0.001), with an intermediate effect value of 0.122, representing 32.35% of the total effect.

**Conclusion:**

Academic pressure is associated with an increase in self-regulatory fatigue, mediated by social support. Educational administrators should pay attention to the social support and resource supplement of nursing students, the adjustment and compensatory development of nursing students’ physical and mental resources, the advancement of nursing students’ internal resource adjustment, and the reduction of their self-regulatory fatigue.

## Background

Stress, which can cause a variety of physiological changes and reactions, is defined as “the incapacity to cope with perceived (actual or perceived) hazards to mental, physical, emotional, and spiritual welfare [1].” College can be a stressful time for young adults transitioning into adulthood [2]. According to several factors, including environmental changes, academic demands, higher education programs, competition with other students, the transition to independence, and failure, academic stress is currently the leading cause of stress for college students [3, 4]. It may even be higher than in high school. A public opinion poll found that approximately 80% of college students around the world said they were under academic stress [5].

The World Health Organization defines academic stress as “the perception by certain students of events in the learning process as a threat to themselves, resulting in a range of undesirable behaviors” (e.g., worry, anxiety, or fear). It has been demonstrated that persistent learning stress can cause symptoms and issues such as low energy, depression, trouble concentrating, irritation, stress, and physical discomfort [6]. According to a poll of 27,343 college students who were under academic stress, 23% of college students reported feeling very stressed out and 91% reported having bad academic things happen to them in the previous 6 months [7]. This indicates that college students have reached a degree of academic stress that warrants considerable inquiry.

Chinese nursing students (referred to in this paper as “nursing students”) are the reserve army of China’s nursing career and have a significant impact on the growth of China’s nursing workforce. However, as a result of the impact of traditional Confucianism, the Chinese educational system is frequently based on a number of different examinations or tests. This results in a high-stress atmosphere for Chinese students [8]. Academic burnout was observed to affect up to 39.29% of a sample of 733 nursing students [9]. In a separate study, 58.34% of the students were in a moderate state of stress, and the Academic Stress Scale revealed that tests were the primary source of stress among students [10]. Therefore, adequate consideration should be given to the academic stress situation of nursing students, as excessive academic stress can expose them to additional detrimental effects.

Self-control can be defined as “the mental process through which a person overcomes, inhibits, or alters his or her impulses, thoughts, emotions, and behaviors in order to adhere to social norms and personally accepted global goals” [11]. self-regulatory fatigue occurs when people engage in self-control despite diminished volitional activity capacity or willingness, which can result in a decline in individual management behavior and confidence in one’s own ability to do so [12]. In the model of self-regulatory strength, all forms of self-regulation draw from the same finite internal resource or energy pool, called self-regulatory strength. To rephrase, a person’s capacity for self-control is finite, much like the “power” of their muscles. It can be challenging for people to practice further self-control when they make an effort but find that their behaviors use a significant amount of their own regulatory resources [13, 14].

This definition indicates that when nursing students are experiencing a great deal of academic pressure, their own resources continue to be depleted, their physical and mental compensation is not timely, and, as a possible consequence, their learning efficiency decreases, they experience depression, anxiety, fear, and other adverse psychological reactions, and they may even engage in a number of risky behaviors. However, the connection between academic stress and self-regulatory fatigue in nursing students has not been studied.

Social support can be characterized as support that boosts an individual’s psychological drive and provides an emotional, physiological, and cognitive contribution, and that comes from family, friends, and institutions [15]. It has recently been established that social support plays a significant role in the self-regulatory process of tiredness management and is an intrinsic incentive for individual resource recovery [16, 17]. Studies have also shown that social support has a protective effect on mental health in stressful conditions and is a powerful predictor of overall health and well-being that can directly alter psychological processes such as evaluation, mood, and emotion [18]. Therefore, this study presents the idea that social support can successfully serve as a mediator between academic stress and self-regulatory fatigue.

Considering the importance of academic stress, social support, and self-regulatory fatigue for the healthy physical and psychological development of Chinese undergraduate nursing students, we thoroughly investigated the levels of academic, social, and self-regulatory fatigue among a group of Chinese nursing majors. The purpose of this study was to examine the relationship between these three variables, as well as the effects of academic stress on social support and self-regulatory fatigue, and the mediating role of social support between the other two variables, in order to provide empirical support for educational administrators to take effective, comprehensive measures to address self-regulatory fatigue among nursing students from the perspectives of social support and resource suppleness.

## Object and method

We conducted a cross-sectional study at two higher education institutions in Heilongjiang Province, China. A structured online questionnaire was used to collect data for building and testing a model of how nursing students deal with their self-regulatory fatigue.

### Object

Participants were nursing students in grades 1 through 4. The inclusion criteria were as follows: students who comprehended the purpose of the study and volunteered to take part were considered for inclusion. This study excluded subjects who did not respond to one or more questions.

The minimum sample size for structural equation modeling should be more than 10 times the number of paths to estimate in the model [19], so the minimum sample size for this study was 130 cases, and considering a deformation rate of 10%, the sample size for the study should be 140 cases.

### Data collection method

The researcher modified the questionnaire to be compatible with the Questionnaire Star platform (https://www.wjx.cn/vj/PRuTQS0.aspx). On the introductory screen, the survey’s objective, significance, and projected completion time were described. Six nursing students were picked at random for the pre-survey, and the length of the questionnaire was between 5 and 10 minutes, with a reasonable number of participants for each. With the approval of the grade teachers. We recovered 1703 questionnaires and discarded 906 invalid ones, for a valid recovery percentage of 46.80%. Exclusion criteria for invalid questionnaires include:(I) questionnaires with clearly regular responses, such as selecting the same option for all entries;(II) questionnaires with missing items; and (III) questionnaires completed in less than 5 minutes with inconsistent and logically contradictory responses.

### Ethics approval and consent to participate

The Ethics Committee of the Second Affiliated Hospital of Harbin Medical University approved this work (KY2022–119). Prior to the commencement of the investigation, participants were told of the objectives and methodology of the study; informed consent was acquired; and they were advised that participation was optional and that their replies would be treated anonymously. All methods comply with predetermined standards.

### Survey tools

- **General Information Questionnaire.** Designed by the researcher, the variables gender, age, educational background, and grade are included.
- **Self-regulated Fatigue Scale.** The Self-regulated Fatigue Scale was compiled by Wang et al. [20] Included were 16 items in 3 dimensions: cognitive control (6 items), emotional control (5 items), and behavioral control (5 items). The Likert5 grade scoring method is adopted (1 point for very different opinions, 2 points for different opinions, 3 points for uncertainty, 4 points for agreement, and 5 points for very agreement). The total score range is 16 to 80 points. The higher the score, the heavier the self-regulatory fatigue level. In this study, the Cronbach’s alpha coefficient of the scale was 0.877, the KMO number was 0.911, and the Bartlett’s value was 4187.029 (*p*<0.0001), indicating excellent reliability and validity.
- **Social Support Rating Scale.** The questionnaire is based on Xiao et al. [21]. Sun and colleagues [22] modified some items in the scale, changed “spouse” to “lover” and removed the item “children”. The scale includes 10 items in 3 dimensions: objective support (3 items), subjective support (4 items) and utilization of support (3 items). The scoring principle of the scale is that items 1 to 4 and 8 to 10 are scored 1, 2, 3, and 4 respectively. Article 5: total score of 4 items. In Articles 6 and 7, if the answer is “no source”, 0 points will be given. If the answer is “the following sources,” several points will be given. The total score ranges from 10 to 65. The higher the score, the more social support the nursing students perceive. In this study, the Cronbach’s alpha coefficient of the scale was 0.744, the KMO number was 0.686, and Bartlett’s value was 7332.850 (*p*<0.0001). Validity and dependability were adequate.
- **Academic Stress Scale.** The 2007 questionnaire created by Tian LAN and Deng Qi [23] was chosen. The scale consists of 42 items distributed across 7 dimensions: pressure on study prospects (9 items), pressure on academic competition (8 items), pressure on study effectiveness (8 items), pressure on study atmosphere (4 items), pressure on class workload (5 items), pressure on study conditions (4 items), and pressure on family expectations (4 items). It utilized a 5-point Likert scale (1 point for not at all, 2 points for a little, 3 points for the majority, 4 points for significantly, and 5 points for entirely). The score range is 51–255, and the higher the score, the greater the learning strain. In this study, the Cronbach’s alpha coefficient of the scale was 0.967, the KMO number was 0.970, and the bartlett’s value was 22038.818 (*p*<0.0001). These values indicate strong reliability and validity.

### Statistical method

The IBM SPPS Statistics and AMOS Graphics packages were used for data analysis in this study.

Using descriptive statistics, Pearson was initially employed to examine the link between academic stress, social support, and self-regulatory fatigue (mean, standard deviation, skewness, and kurtosis).

Path analysis was performed using the structural equation model (Amos) to examine the relationship among variables. Skewness and kurtosis were first calculated based on normality. In this study, the skewness of the variables was found to vary between − 0.448 and 0.657, while the kurtosis values varied between − 0.364 and 1.042. For normal distribution, skewness and kurtosis values within±2 range are considered acceptable [24]. The results show that the data from this study is applicable to AMOS analysis. The goodness of fit of the structural model was calculated using the comparative fit index (CFI), the goodness of fit index (GFI), the normative fit index (NFI), the root mean square error of approximation (RMSEA), and the relative chi-square(χ^2^) analysis of the model’s goodness of fit/(df). To ensure the significance of indirect and direct effects of variables included in Amos, 95% confidence intervals were applied.

The following adjustment rates were considered: the incremental rates (IFI, incremental fit index; CFI, comparative fit index; and TLI, Tucker Lewis index) had to show scores above 0.95; the error rates root mean square error of approximation (RMSEA) and standardized root mean square residual (SRMSR) were considered acceptable values equal to or less than 0.06 and 0.08, respectively; and finally, the values χ^2^/df, which was considered an acceptable value below 3.

We examined the direct and indirect impacts of self-regulatory fatigue and social support on academic stress using the mediation model. In the mediation model, the effect of variables as mediators in explaining the relationship between independent and dependent variables was studied.

## Result

### Common method deviation inspection

The examination was conducted using Harman’s single-factor test. All variables were included to test the results of the non-rotating factor analysis. From the results, it was found that there were 12 factors with eigenvalues greater than 1, and the variation explained by the first factor was 20.895%, lower than the critical one of 40%. Therefore, there are no serious common methodological deviations in this study.

### Participant characteristics

A total of 797 participants in the sample included 643 (80.7%) women and 154 (19.3%) men. Age distribution: 124 (15.6%) were 16–18 years old, 649 (81.4%) were 19–21 years old, and 24 (3.0%) were 22–24 years old. In terms of education, 27 (3.4%) were undergraduates and 770 (96.6%) were specialists. In terms of grades, 741 (93%) in the first grade, 9 (1%) in the second, 40 (5%) in the third, and 7 (0.9%) in the fourth.

### Social support, academic stress and self-regulatory fatigue scores of nursing students

The total score of academic stress of nursing students was 111.28 ± 29.38 (skewness = − 0.448; kurtosis = 0.380), the total score of social support was 37.87 ± 6.70 (skewness = 0.069; kurtosis = 0.054), and the total score of self-regulatory fatigue was 45.53 ± 5.55 (skewness = 0.358; kurtosis = 0.526). See Table 1 for details.

**Table 1:** Scores of social support, academic stress and self-regulatory fatigue of nursing students (*n* = 797)

Variable	Min	Max	Average	Standard	Skewness	Kurtosis
**Total academic stress score**	42.00	209.00	118.28	29.38	−0.448	0.380
Learning prospect pressure	9.00	45.00	26.23	6.29	-0.379	0.379
Academic competitive pressure	8.00	40.00	22.86	6.20	−0.360	0.375
Learning effectiveness pressure	8.00	40.00	23.41	6.45	−0.417	0.308
Learning atmosphere pressure	4.00	20.00	10.05	3.43	−0.190	− 0.364
Schoolwork burden pressure	5.00	25.00	14.50	4.52	−0.286	0.035
Learning condition pressure	4.00	20.00	10.97	3.43	−0.372	− 0.091
Family expectation pressure	4.00	20.00	10.26	3.42	−0.268	−0.276
**Total score of social support**	15.00	59.00	37.87	6.70	0.069	0.054
Subjective support	8.00	28.00	21.12	3.85	-0.308	−0.308
Objective support	3.00	20.00	9.21	2.76	−0.657	−0.454
Utilization of support	3.00	12.00	7.54	1.95	0.342	0.199
**Total score of self-regulatory fatigue**	29.00	65.00	45.53	5.55	0.358	0.526
Cognitive control	10.00	29.00	19.68	2.38	−0.152	1.042
Behavior control	7.00	24.00	12.76	2.83	0.472	0.150
Emotion control	5.00	24.00	13.10	3.10	0.294	−0.036

### Correlation among social support, academic stress and self-regulatory fatigue of nursing students

Academic stress was negatively correlated with social support (*r* = − 0.236, *p* < 0.01), academic stress was positively correlated with self-regulatory fatigue (*r* = 0.257, *p* < 0.01), and academic stress was negatively correlated with self-regulatory fatigue (*r* = − 0.190, *p* < 0.01). The results are shown in Table 2.

**Table 2:** Correlation analysis results of social support, academic stress and self-regulatory fatigue of nursing students (*n* = 797)

Variable	Social support	Academic pressure	Self-regulatory fatigue
Social support	1		
Academic pressure	−0.236^a^	1	
Self-regulatory fatigue	−0.190^a^	0.257^a^	1

^a^Significant at 0.01 level (double tailed)

### A test of the mediating influence of social support on the relationship between academic stress and self-regulatory exhaustion in nursing students

**Fig. 1:**
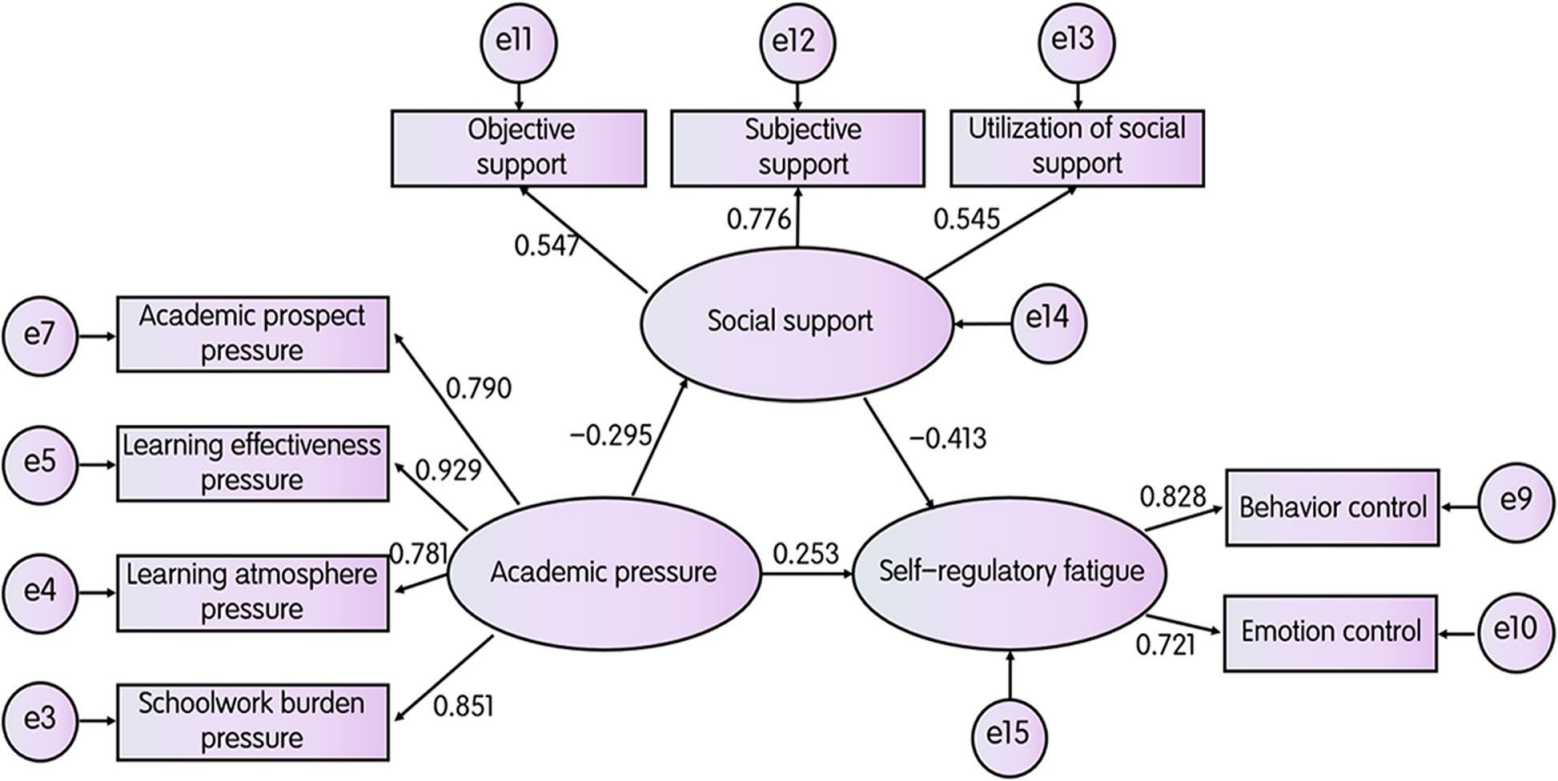
A mediated model of social support mediating academic stress and self-regulatory fatigue in nursing students

In this study, the nonparametric percentile bootstrap program with Amos bias correction was used to test the significance of the mediating effect. 5000 samples were randomly selected from the original sample (*n* = 797) to reduce class I errors in statistical reasoning caused by data. According to the theoretical assumptions of this study, during the modeling process, the academic pressure of nursing students (seven latent variables: learning prospect pressure, academic competition pressure, learning effectiveness pressure, learning atmosphere pressure, schoolwork burden pressure, learning condition pressure, and family expectation pressure) was taken as the predictive variable, and social support (three latent variables: objective support, subjective support, and utilization of support) was taken as the intermediary variable. Self-regulated fatigue (three latent variables: cognitive control, behavioral control, and emotional control) is used as the effect variable to establish the intermediary effect structural equation model, and the maximum likelihood ratio method is used to establish the correlation between residuals according to the correction indicators given by the model, so as to appropriately improve the model fitting, as shown in Fig. 1. Each fitting index of the structural equation model meets the ideal standard, and the model is well adapted, as shown in Table 3.

**Table 3:** Structural equation model fitting index (*n* = 797)

Project	χ^2^	CMIN/df	GFI	AGFI	RFI	NFI	CFI	IFI	TLI	RMSEA
Modified fitting index	78.294	3.262	0.979	0.960	0.962	0.974	0.982	0.982	0.973	0.053
Acceptable standards	–	< 5	> 0.9	> 0.9	> 0.9	> 0.9	> 0.9	> 0.9	> 0.9	< 0.08

*CMIN/df* Maximum likelihood ratio χ^2^ values / degrees of freedom, *GFI* Goodness of fit index, *AGFI* Adjusted goodness of fit index, *RFI* Relative fitness index, *NFI* Standard fit index, *CFI* Comparative fit index, *IFI* Value added index, *TLI* Nonstandard fit index, *RMSEA* Mean square sum square root of progressive residuals

### Estimated parameters among academic stress, social support and self-regulatory fatigue of nursing students

The regression coefficients of social support in the two paths of nursing students’ academic stress (*B* = -0.295, *p* < 0.001) and self-regulatory fatigue (*B* = -0.413, *p* < 0.001) were significant. Therefore, the mediating effect of social support was significant. Academic stress of nursing students has a positive predictive effect on self-regulatory fatigue (*B* = 0.253, *p* < 0.001). See Table 4 for details.

**Table 4:** Estimated parameters and 95% CI between academic stress, social support and self-regulatory fatigue of nursing students (*n* = 797)

Project	Estimate	SE	Z(sig.)	confidence interval	
				Boot CI Upper	Boot CI Lower
self-regulatory fatigue < −-- academic stress	0.253	0.052	4.865(*p*<0.001)	0.144	0.348
social support < −- academic pressure	−0.295	0.044	6.705(*p*<0.001)	−0.379	− 0.211
self-regulatory fatigue < −- social support	− 0.413	0.055	7.509(*p*<0.001)	−0.514	− 0.300

Standardized Regression Weights

### Calculation of total effect, direct effect and intermediary effect

Social support played a part in the mediating effect between nurses’ work stress and self-regulatory fatigue, and the mediating effect value was (− 0.295 × − 0.413) = 0.122. The total effect (intermediary effect + direct effect) is (0.122 + 0.253) =0.375. The ratio of intermediary effect to total effect is 32.35%. See Table 5 for details.

**Table 5:** Overall effect, direct effect, and intermediary effect breakdowns

	Effect value	Boot CI Upper	Boot CI Lower	Relative effect value
Total effect	0.375	0.277	0.456	
Direct effect	0.253	0.144	0.348	67.65%
Mediating effect	0.122	0.079	0.177	32.35%

The bootstrap method estimates the standard error of indirect effects and the lower and upper limits of 95% confidence intervals

## Discussion

### Nursing students’ levels of social support, academic stress, and self-regulatory fatigue

Consistent with the findings of McLean [25] and Wang [26], the total score of social support for nursing students in this study was 37.87 ± 6.70, indicating a moderate level according to the scale rating criteria. This may be because Chinese universities now have more professors and policies designed to provide college students with greater academic help and guidance. In addition, educational institutions in China today provide a broader range of training and practical activities, both inside and outside of the classroom, giving students greater opportunity for social activities and a sense of accomplishment and support [26]. Among the three categories of social support, it is important to note that subjective support scores were high, while support usage ratings were relatively low, indicating that certain students were less able to utilize the available social support resources. It is recommended that educational administrators mobilize adequate social support forces through active communication platforms, magnetic learning settings, family connection, and psychological counseling.

### Nursing students are experiencing moderate to high levels of academic pressure

In this study, the total academic stress score of nursing students was 118.28 ± 29.38 with a total score of 140, which is high and similar to Wang et al.’s finding [27]. According to the Selameab [28] report, 56.3% of nursing students felt unmanageable stress in the preceding 30 days. As mentioned in the paper, learning and life, imbalances in leisure, sleep issues, competitive emotions, rigorous periodic testing, fear of failure, and high parental expectations all contribute to higher academic stress [29]. Moreover, nursing is a unique profession that focuses on protecting the health and lives of patients, particularly in a “patient-centered” work environment. Due to the nature of the position and the current training requirements, nursing students must not only master the professional competencies of serving patients but they must also actively study interpersonal communication courses to enhance their ability to handle clinical emergencies, which increases the academic pressure on nursing students. Administrators of nursing education should therefore pay close attention to the academic performance of nursing students and manage their academic stress in a timely manner by enhancing psychological communication channels and cultivating psychological diversion skills.

### Nursing students’ self-regulatory fatigue is at a moderate degree

The total self-regulatory fatigue scores of nursing students in the present study were moderate at 45.53 ± 5.55, similar to the previous findings [30]. According to studies, prolonged, high-intensity role stress can exacerbate mental health damage and lead to emotional exhaustion [31, 32]. The self-regulatory power model proposed by Baumeister suggests that the implementation of self-control is dependent on limited energy resources, just as human muscles fatigue after a certain period of exercise. Self-control behavior will lead to the depletion of psychological resources, which will, in the short term, hinder the implementation of future self-control behaviors [33–35]. Under the influence of high academic pressure, nursing students require long periods of physical and energy effort, and in the continuous loss of resources, they will experience psychological discomfort and accumulated stress, and their self-control will be gradually depleted, leading to academic exhaustion, emotional fatigue, and even interpersonal aggression and practice errors, as well as other failures of self-mastery. Therefore, educational administrators should focus on enhancing the psychological training of nursing students and supplementing their internal resources to facilitate their own timely and effective physical and mental adjustment.

### Correlations analysis of social support, nursing students’ academic stress, and self-regulatory fatigue

This study shows that perceived support was significantly and negatively correlated with nursing students’ academic stress (*r* = − 0.236, *p* < 0.01), which is consistent with the findings of Tian et al. [36], who found that good support can increase a student’s sense of belonging, decrease academic burnout, and motivate them to learn, thereby reducing their academic stress. Secondly, the study revealed that social support was significantly and negatively related to self-regulatory fatigue (*r* = − 0.190, *p* < 0.01), which is consistent with the findings of my previous study on nursing staff, which indicated that social support can somehow compensate for the depletion of emotional resources and thus mitigate the negative utility of emotional depletion [37]. In addition, this study revealed a significant and positive correlation between self-regulatory fatigue and academic stress among nurses (*r* = 0.257, *p* < 0.01). Consequently, it suggests that the higher the academic stress and the lower the social support of nursing students, the greater their self-regulatory fatigue scores and susceptibility to self-regulatory fatigue.

### Path analyses of the effects of social support and academic stress on self-regulatory fatigue in nursing students

The results of this study showed that the path coefficient of social support in the effect of academic stress on nursing students (*B* = -0.295, *p* < 0.001) and the path coefficient of social support in the effect of self-regulatory fatigue (B = -0.413, *p* < 0.001) both reached significant levels, while the path coefficient of academic stress in the effect of self-regulatory fatigue of nursing students (*B* = 0.253, *p* < 0.001), which indicated that social The mediating effect of social support was significant and partially mediated between nursing students’ academic stress and self-regulatory fatigue with a mediating value of (− 0.295 × − 0.413) =0.122. As mentioned in the study, social support is often considered an important compensator, buffering individual psychological responses when faced with challenging circumstances and is considered a moderator of the relationship between stressors and psychological outcomes [38]. Thus, educational administrators should focus on “energy depletion” and “resource replenishment” and pay attention to the regulation and compensatory development of nursing students’ physical and psychological resources.

### Strengths and limitations

This looked into the connection between academic stress, social stress, and self-regulatory fatigue in nursing students, which is one of its greatest strengths. To our knowledge, this is the first study to simultaneously examine these three variables among student nurses. The present study also revealed a moderate level of self-fatigue among nursing students, which should be paid attention to and has been described infrequently in previous research. Importantly, this study examines the social support pathway surrounding the physical and mental adjustment of current nursing students. Therefore, it is recommended that educational administrators in the future provide strategies such as peer strength [39], emotional support from families [40, 41], and supportive teachers [42, 43] to facilitate the recovery of physical and mental sources of strength for students through effective counseling and supportive training [44–46]. The low recovery rate of 46.80% in this study can be partly blamed on the fact that students were busy with their studies and didn’t have time to fill out the scale.

Obviously, every study will have its limits. First, our study is a cross-sectional investigation that can only explain the correlation between academic stress, social support, and self-regulatory exhaustion among nursing students, not their causal link. Ideally, future research should involve national and multicenter investigations. Second, the questionnaire was selected using a convenience sampling method, which may have influenced the sample’s representativeness due to probable selection bias. Nonetheless, we acquired a sample of 797 respondents, and this large sample partially compensated for this shortcoming. Thirdly, the majority of participants in the general demographic characteristics were female and between the ages of 19 and 21, although these demographic aspects were not adjusted for in the model tests conducted by the authors, which could have influenced the results. In future studies, we will think about how adjusting for demographic factors might affect the results.

### Summary

In addition to demonstrating the effect of nursing students’ academic stress on self-regulatory fatigue, this study also reveals the mediating role of social support in the interaction between nursing students’ academic stress and self-regulatory fatigue, which has implications for widening and deepening research on the relationship between nursing students’ academic stress and self-regulatory fatigue. In particular, with respect, it provides a complementary resource perspective for nursing education administrators to alleviate the students’ self-regulatory fatigue.

## Data Availability

Data sets used and / or analyzed during the current study may be obtained from the appropriate authors upon reasonable request.
